# Bioenergetics and Autophagic Imbalance in Patients-Derived Cell Models of Parkinson Disease Supports Systemic Dysfunction in Neurodegeneration

**DOI:** 10.3389/fnins.2019.00894

**Published:** 2019-09-10

**Authors:** Ingrid González-Casacuberta, Diana Luz Juárez-Flores, Constanza Morén, Gloria Garrabou

**Affiliations:** ^1^Muscle Research and Mitochondrial Function Laboratory, Cellex-IDIBAPS, Faculty of Medicine and Health Sciences-University of Barcelona, Internal Medicine Service-Hospital Clínic of Barcelona, Barcelona, Spain; ^2^CIBERER-U722, Madrid, Spain

**Keywords:** neurodegeneration, mitochondria, autophagy, Parkin, LRRK2, fibroblasts

## Abstract

Parkinson’s disease (PD) is the second most prevalent neurodegenerative disorder worldwide affecting 2–3% of the population over 65 years. This prevalence is expected to rise as life expectancy increases and diagnostic and therapeutic protocols improve. PD encompasses a multitude of clinical, genetic, and molecular forms of the disease. Even though the mechanistic of the events leading to neurodegeneration remain largely unknown, some molecular hallmarks have been repeatedly reported in most patients and models of the disease. Neuroinflammation, protein misfolding, disrupted endoplasmic reticulum-mitochondria crosstalk, mitochondrial dysfunction and consequent bioenergetic failure, oxidative stress and autophagy deregulation, are amongst the most commonly described. Supporting these findings, numerous familial forms of PD are caused by mutations in genes that are crucial for mitochondrial and autophagy proper functioning. For instance, late and early onset PD associated to mutations in Leucine-rich repeat kinase 2 (*LRRK2*) and Parkin (*PRKN*) genes, responsible for the most frequent dominant and recessive inherited forms of PD, respectively, have emerged as promising examples of disease due to their established role in commanding bioenergetic and autophagic balance. Concomitantly, the development of animal and cell models to investigate the etiology of the disease, potential biomarkers and therapeutic approaches are being explored. One of the emerging approaches in this context is the use of patient’s derived cells models, such as skin-derived fibroblasts that preserve the genetic background and some environmental cues of the patients. An increasing number of reports in these PD cell models postulate that deficient mitochondrial function and impaired autophagic flux may be determinant in PD accelerated nigral cell death in terms of limitation of cell energy supply and accumulation of obsolete and/or unfolded proteins or dysfunctional organelles. The reliance of neurons on mitochondrial oxidative metabolism and their post-mitotic nature, may explain their increased vulnerability to undergo degeneration upon mitochondrial challenges or autophagic insults. In this scenario, proper mitochondrial function and turnover through mitophagy, are gaining in strength as protective targets to prevent neurodegeneration, together with the use of patient-derived fibroblasts to further explore these events. These findings point out the presence of molecular damage beyond the central nervous system (CNS) and proffer patient-derived cell platforms to the clinical and scientific community, which enable the study of disease etiopathogenesis and therapeutic approaches focused on modifying the natural history of PD through, among others, the enhancement of mitochondrial function and autophagy.

## Parkinson’s Disease

Parkinson’s disease (PD) is the most common movement disorder and the second most frequent neurodegenerative disease affecting more than 6.5 million people worldwide (Teves et al., 2017), representing 2–3% of the population over 65 years (Connolly and Lang, 2014; Poewe et al., 2017). As the global life expectancy increases, the number of people with PD is expected to rise by more than 50% in 2030, constituting an important burden for public health (Kalia and Lang, 2015).

Although it was already known in ancient India under the name of “Kampavata,” PD was first described in 1817 by James Parkinson in the *Essay on the Shaking Palsy* and later on refined and expanded by Jean-Martin Charcot who named the disorder “malaldie de Parkinson”(Kempster et al., 2007; Corti et al., 2011; Goetz, 2011). Despite PD was described more than two centuries ago, the conceptualization of the disease continues to evolve and it is now recognized as a systemic disease with multiple layers of complexity.

**FIGURE 1:**
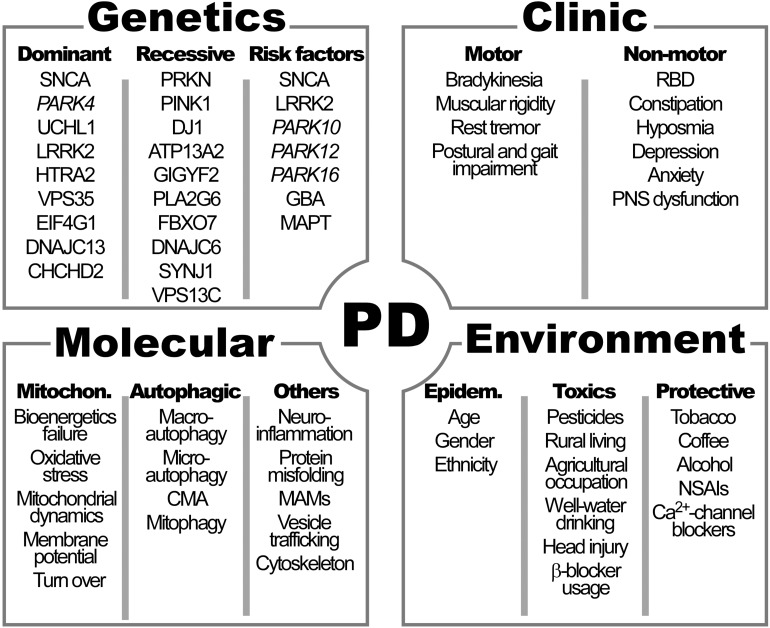
Clinical manifestations and genetic, molecular and environmental factors characteristics of Parkinson’s disease (PD). See Table 1 for the names of genes. RBD, rapid eye movement sleep behavior disorder; PNS, peripheral autonomic nervous system; CMA, chaperon-mediated autophagy; MAMs, mitochondrial associated membranes; NSAIs: non-steroidal antinflammatory drugs; Ca^2+^, calcium.

The cardinal symptoms of PD described by James Parkinson in 1817 and then refined by Jean-Martin Charcot, include bradykinesia, muscular rigidity, rest tremor and postural and gait impairment (Goetz, 2011) (Figure 1).

Pathologically, PD is a complex neurodegenerative disorder characterized by the prominent death of dopaminergic neurons (DAn) in the *substantia nigra* (SN) *pars compacta* (SNpc) located in the mesencephalon and the consequent striatal dopamine (DA) deficit that leads to the classical motor symptoms of the disease (Kalia and Lang, 2015). In addition to the loss of DAn, another hallmark of PD is the presence of intraneuronal inclusions in the soma of the remaining DAn. These inclusions, named Lewy bodies (LB) as well as Lewy neurites (LN), are collectively referred as Lewy pathology (LP). LB are round eosinophilic inclusions mainly formed by insoluble α-synuclein aggregates as well as ubiquitin and other proteins (Shults, 2006). The aggregation of these misfolded proteins has been shown to be common to PD, dementia with LB, and multiple system atrophy (Goedert et al., 2013).

### Clinical Features

The dramatic loss of DAn in the SNpc, even in early stages of the disease, suggests that the degeneration in this region starts long before the motor symptoms appear (Poewe et al., 2017). In this context, PD is considered to occur in three stages: preclinical PD, when the neurodegeneration has started but no clinical signs or symptoms are present; premotor or prodromal PD, when clinical signs and/or symptoms are present but are insufficient to establish a diagnosis of PD; and clinical PD, when the diagnostic criteria are met (Kalia and Lang, 2015, 2016). Non-motor features are frequently present in the prodromal phase of the disease, which can last for 20 years or more, and involve a multitude of non-motor features including rapid eye movement (REM) sleep behavior disorder (RBD), constipation and hyposmia, as well as depression and anxiety (Poewe, 2008).

In this scenario, it is now widely accepted that PD is not a movement disorder simply induced by the loss of the DAn in the SNpc. The SN is not the only damaged region in PD, nor the first affected one. Brain sites other than the SN, such as the cerebral cortex and the limbic system, have also been reported to be impaired in patients during the presymptomatic phase (Dickson, 2018). In fact, several studies have shown that the degenerative process in PD is much more extensive and affects not only the central nervous system (CNS) but also the peripheral autonomic nervous system (PNS) and the organs outside the brain that the latter innervates (Braak et al., 2004). PNS dysfunction underlies the presence of some of the specific non-motor features that appear in the prodromal phase of PD and remain present over the course of the disease (Jain, 2011). In line with this, LP is not restricted to the brain but has also been encountered in the spinal cord and PNS including the vagus nerve, sympathetic ganglia, cardiac plexus, enteric nervous system (ENS), salivary glands, adrenal medulla, cutaneous and sciatic nerves (Tolosa and Vilas, 2015).

### Risk Factors

Parkinson’s disease was thought to be primarily caused by environmental factors, but research reveals that the disease develops from a complicated interplay of ageing, genetics and environment. In fact, the vast majority of cases occur sporadically and genetic forms of the disease account for about 10% of patients (Klein and Westenberger, 2012).

In general, the average age of onset of PD is the late fifties, with a broad range from <40 to >80 years of age depending, among others, on its pattern of inheritance. Young-onset PD is commonly defined by an age of onset <45 years and >10% of these cases have a genetic basis; the proportion of genetically defined cases rises to >40% of those with disease onset before 30 years of age (Alcalay et al., 2010; Marder et al., 2010).

#### Non-genetic Risk Factors

The greatest risk factor for the development of neurodegenerative diseases, including PD, is ageing (Kalia and Lang, 2015). Incidence increases nearly exponentially from the sixth to the ninth decade of life by 5–10 fold (Kalia and Lang, 2015; Poewe et al., 2017). Total global prevalence is 0.3% and rises with age up to 3% in those >80 years of age (Poewe et al., 2017) (Figure 1). Many lines of evidence suggest that some molecular pathways including mitochondrial dysfunction, oxidative stress and abnormal cell wasting clearance (autophagy) have a central role in both, physiological aging and age-related neurodegenerative diseases, such as PD (Lin and Beal, 2006). This may be especially relevant in neurons, due to its post-mitotic nature and scarce replacement, that prone them to store defects as they age. Accelerated or healthy aging and all factors responsible of modulating brain fragility play a major role in PD development, together with genetic and environmental factors.

Gender has also been reported to be a risk factor for PD, with approximately a 3:2 male-to-female ratio. Sex hormones have been proposed to play a neuroprotective role in the disease. In case of female hormones, the antioxidant capacity of estradiol, for instance, has been proposed to prevent neurodegeneration (Aguirre-Vidal et al., 2017). Interestingly, estradiol has also been demonstrated to activate metabolic signaling by regulating mitochondrial function, emerging as protective hormone in case of bioenergetic deficits (Pozdniakova et al., 2018). The neuroprotecting role of progesterone is also being evaluated (Bourque et al., 2019). Alternatively, gender associated differences could also be associated to sex-associated genetic mechanisms, to specific differences in exposure to environmental cues or to the contribution of inequality in health care (Kalia and Lang, 2015). Interestingly, in a few populations, including one study from Japan, no differences in gender, and even increased prevalence in females, was observed (Kusumi et al., 1996). The explanation for equal gender PD prevalence in these populations remains elusive. Dietetic, socio-cultural, economic or even hormonal or molecular characteristics of Japanese population (as particular mitochondrial DNA haplogroups) may justify gender equivalence in PD development.

Other risk factors for PD are directly associated with environmental features are pesticide exposure, rural living, agricultural occupation, well-water drinking, prior head injury and β-blocker use. A special mention must be done for exposure to 1-methyl-4-phenyl-1,2,3,6-tetrahydro pyridine (MPTP), with similar structure of some herbicides that increase the risk of PD, due to its historical importance. Since its incidental discovery in drug abusers after inadvertent self-administration, MPTP has been widely used to induce PD in animal models (Zhang et al., 2019). MPTP is lipid-soluble molecule that penetrates the blood–brain barrier and, once converted to its oxidized product (MPP+), interferes with mitochondrial respiration. MPP+ acts specifically in mitochondrial complex I, such as other toxic PD inducers (including rotenone and pesticides). Blockade of mitochondrial respiration has three main toxic consequences to cells: the inhibition of ATP generation and associated bioenergetic failure, the derived elevation of intracellular Ca^2+^ that promotes cell death and the promotion of oxidative stress responsible of cell damage, all hallmarks of PD pathogenesis.

In contrast, tobacco smoking, coffee drinking, non-steroidal anti-inflammatory drug use, calcium channel blocker intake, and alcohol consumption have been associated with a decreased risk of PD development (Kalia and Lang, 2015).

Additionally, the incidence of PD seems to vary within different ethnicities. The prevalence of PD is high in Ashkenazi Jews of Israel, Inuit, Alaska native and native American populations (de Lau and Breteler, 2006; Poewe et al., 2017) and only one study reports that PD is more common in Hispanic and non-Hispanic whites compared to African Americans and Asian in United States (Van Den Eeden et al., 2003). The higher incidence of PD in these populations has been classically attributed to specific genetic burden.

#### Genetic Risk Factors

There are two different classes of genetic contributions to PD: gene mutations directly associated to genetically inherited forms of PD and genetic variations, indirectly accounting for risk factors of disease.

With respect to the first, the existence of heritable forms of PD was originally established through the discovery in 1997 that mutations in *SNCA*, the gene encoding for the α-synuclein protein, caused PD and the demonstration that α-synuclein was the major component of LB (Corti et al., 2011). Since then, the list of mutations causing monogenic types of PD continues to grow associated either to dominant or recessive inherited forms of PD (Table 1 and Figure 1). Currently 8–10% of cases are familial (result from a genetic alteration leading to PD). They are caused by a subset of *locus* usually encoded by the prefix PARK and a number referring to their order of discovery. Those mutations affecting genes with an autosomal recessive pattern of inheritance usually result in early onset cases of PD, while mutations affecting genes with dominant autosomal inheritance usually cause forms of PD that resemble late-onset idiopathic PD.

**TABLE 1:** Classification of genes associated with familial forms of Parkinson’s disease.

**Gene name**	**Gene symbol**	**Gene locus**	**Protein**	**Protein function and cell pathway governated**	**Onset of disease**
**Autosomal dominant inheritance Parkinson’s disease**					
*SNCA*	PARK 1 or PARK4	4q22.1	Alpha-synuclein	Synaptic vesicles trafficking	Early
Unknown	PARK3	2p13	Unknown	Unknown	Late
*UCHL1*	PARK5	4p13	Ubiquitin C-terminal hydrolase L1	Proteasome system	Late
*LRRK2*	PARK8	12q12	Leucine-rich repeat kinase 2	Autophagy processing	Late
*HTRA2*	PARK13	2p13.1	HtrA serine peptidase 2	Mitophagy development	Unknown
*VPS35*	PARK17	16q12	Vacuolar protein sorting 35	Endosome regulation	Late
*EIF4G1*	PARK18	3q27.1	Eukaryotic translation initiation factor 4 gamma 1	Protein translation	Late
*DNAJC13*	PARK21	3q22.1	DnaJ heat shock protein family (Hsp40) member C13	Endosome regulation	Late
*CHCHD2*	PARK22	7p11.2	Coiled-coil-helix-coiled-coil-helix domain containing 2	Mitochondria-mediated apoptosis and metabolism	Late/Early
**Autosomal recessive inheritance Parkinson’s disease**					
*PRKN*	PARK2	6q26	Parkin	Mitophagy development	Early
*PINK1*	PARK6	1p36.12	PTEN-induced putative kinase 1	Mitophagy development	Early
*DJ-1*	PARK7	1p36.23	DJ-1	Mitophagy development	Early
*ATP13A2*	PARK9	1p36.13	ATPase cation transporting 13A2	Lysosomal function	Early
*GIGYF2*	PARK11	2q36-7	GRB10 interacting GYF protein 2	Insulin-like growth factors (IGFs) signaling	Early
*PLA2G6*	PARK14	22q13.1	Phospholipase A2 group VI	Lipids metabolism	Early
*FBXO7*	PARK15	22q12.3	F-box protein 7	Mitophagy development	Early
*DNAJC6*	PARK19	1p31.3	DnaJ heat shock protein family (Hsp40) member C6	Endosome regulation	Early
*SYNJ1*	PARK20	21q22.11	Synaptojanin 1	Endosome regulation	Early
*VPS13C*	PARK23	15q22.2	Vacuolar protein sorting 13 homolog C	Mitophagy development	Early
**Risk factors for developing PD**					
*SNCA*	PARK 1 or PARK4	4q22.1	Alpha-synuclein	Synaptic vesicles trafficking	Early
*LRRK2*	PARK8	12q12	Leucine-rich repeat kinase 2	Cytoskeleton/vesicle transporting/autophagy regulation	Late
Unknown	PARK10	1p32	Unknown	Unknown	Unknown
Unknown	PARK12	Xq21-q22	Unknown	Unknown	Unknown
Unknown	PARK16	1q32	Unknown	Unknown	Unknown
*GBA*	–	1q22	Glucosylceramidase beta	Lysosomal function	Late
*MAPT*	–	17q21.31	Microtubule associated protein tau	Microtubule structure	Sporadic

*Modified from Del Rey et al. (2018).*

The discovery of the molecular pathways orchestrated by proteins encoded by these genes associated with monogenic forms PD, have reinforced the notion that impaired mitochondrial and autophagy homeostasis are key events in disease etiology (Park et al., 2018). In fact, impaired mitochondrial function and autophagy have been directly linked to mutations of at least eleven of the genes associated to familial PD (Table 1). Among them, Leucine-rich repeat kinase 2 (*LRRK2*) and Parkin (*PRKN*) genes emerge amongst the most frequent forms of autosomal dominant and recessive forms of PD, respectively.

With respect to the second, genetic risk factors of PD account for the rest of 90–92% of non-inherited cases of PD, so-called idiopathic. They are caused by the complex interplay of an array of unknown factors, a part from the numerous genetic risk factors of PD (see Table 1): modifying effects by susceptibility alleles, environmental exposures and gene-environmental interactions that may condition gene expression. Some of these genetic risk factors that may conditionate the development of PD are in common to lysosomal storage diseases, mitochondrial pathologies or genes governing autophagic processes.

The definition of genetic and environmental cues in the development of PD is one of the novel areas of study in which growing and coming efforts should be focused and, probably, thanks to the development of new generation sequencing tools, the discovery of novel gens responsible of PD will arise, together with new putative genetic risk factors, thus reducing the number of idiopathic PD cases.

Table 1 includes mutations directly associated to genetically inherited forms of PD (either of dominant or recessive inheritance) and genetic variations indirectly accounting for risk factors of disease. Gene name, *locus* and symbol have been described, together with the protein encoded by the gene and the function it exerts (the cell pathway governated), as well as the kind of PD associated to the genetic mutation or variation (responsible of early, late, sporadic or unknown onset). Among these entities, *LRRK2* and *PRKN* genes emerge amongst the most frequent forms of autosomal dominant and recessive inherited-forms of PD, respectively.

## Models to Study Parkinson’s Disease

A major challenge to study PD is the inaccessibility of the target tissue of the disease (DAn), which is currently only available *post-mortem*. In addition, by the time that clinical symptoms manifest, most of the cells targeted by PD have already been lost (Grosch et al., 2016). Thus, finding models that faithfully recapitulate the events in PD is essential to understand the impaired molecular processes that underlie the disease etiopathogenesis and its progression. In this regard, different experimental *in vitro* and *in vivo* models of study have been consistently used (Blesa et al., 2012; Falkenburger et al., 2016).

### Animal Models

Animal models have allowed the study of PD *in vivo*, partially reproducing the specific pathogenic events and behavioral outcomes of the disease. In fact, after the study of brain necropsies from PD patients, much of the current understanding of the etiology and the pathogenesis of PD has been obtained from the study of neurotoxin-based animal models (Bezard et al., 2013), only recently complemented by experimental approaches targeting genes responsible of the disease. Typical cases of toxic exposure to induce PD in animal models is the use rotenone and MPTP exposition, which reinforced mitochondrial implication in PD. However, toxin-based models rely on acute insults to the nervous system and do not model the slow neural degeneration and development of clinical manifestations characteristic of PD (Westerlund et al., 2010) or the presence of LB, hallmark of the disease, thus raising concerns on the recapitulation of PD pathology.

Later, with the identification of PD-related genes, transgenic models including yeast, *Drosophila melanogaster*, *Caenorhabditis elegans* and murine models have been developed (Blesa et al., 2012) as an alternative to the classical toxin-based ones. These models enabled to gain insights in the molecular events underlying the disease, such as mitochondrial and autophagic deregulation, widely demonstrated in the target tissue of PD. These models have shed light into PD pathogenic processes, but have fallen short in replicating the phenotype and pathology of human disease (Dawson et al., 2010). One of the main drawbacks in the use of animal models to study PD is life span difference between species, which may not allow reproducing age-related events that are relevant to disease pathogenesis. On the other hand, there is an important risk when specifically using invertebrates to study PD as relevant pathogenic factors are vertebrate-specific and may be absent in these models. Finally, most of them do not recapitulate the key clinical and neuropathological features of the disease (as trembling and neuromelanin withdrawal in DAn). Maybe this explains why biomarkers of disease previously verified in murine models and treatments that have shown positive outcomes in these models, have not later been predictive of therapeutic success in humans (Vandamme, 2014). The biological differences between mice and humans may be accountable for this fact, and it is an issue that researchers must be aware of and carefully account for when using these animal models.

In this regard, and probably due to a closer similarity, non-human primates (NHP) have been used to generate the most robust and clinically useful models of PD. The current gold standard animal model of PD is a toxin-based NHP induced model, which shows stable, bilateral clinical features that closely resemble idiopathic PD (Johnston and Fox, 2014) and may even exhibit some features of RBD (Verhave et al., 2011). However, this model does neither recapitulate the major pathophysiological hallmark of idiopathic PD, LB, and its utility for the study of prodromal PD has still not been validated (Barraud et al., 2009).

The development of experimental models to elucidate disease etiology, find novel diagnostic/prognostic biomarkers and assay new therapeutic strategies remains as one of the most challenging gaps in PD research.

The use of toxic or genetic animal models of disease has strengths as reproducing the complex interplay between different neural and non-neural brain cells directly in the target tissue of the disease, and the assay of different therapeutic approaches in physiologic context. Unfortunately, in parallel, animal models fail to recapitulate important hallmarks of PD as clinical and anatomopathological features. Other weaknesses, a part of ethical concerns, high economic and facility costs, are that most of them fail to reproduce the influence of aging, epigenetic, and genetic modifying factors characteristic of PD patients. Novel cell models overcome part of these limitations.

### Cell Models

Immortalized cell lines of neural origin, either animal or human, that can also be subjected to toxin exposition or gene editing, have been widely used to model PD (Obinata, 2007). These cell models have provided consistent and reproducible results. Among their main strengths there is their identical genetic background that confers them a large homogeneity (Carter and Shieh, 2015). Moreover, they represent wide platforms for disease modeling due to low cost maintenance and editing easiness. One example is the human-derived neuroblastoma cell line SHSY5Y that can be used undifferentiated or differentiated to DAn to model PD (Lopes et al., 2010; Alvarez-Erviti et al., 2011).

However, immortalized cell lines are also associated to important weaknesses including the presence of genetic instability or the high rate of glycolytic metabolism which pushes toward the use of patient-derived cell models (Frattini et al., 2015).

The fact that the greatest proportion of PD is of unknown cause and the urgent need to find novel biomarkers at the prodromal phase of the disease has encouraged the development of specific patient-derived models. The advantages of these patient-derived cell models is that they recapitulate PD pathogenicity at an individual basis, thus partially circumventing the drawbacks of animal and established cell line models (Teves et al., 2017). In this context, the use of patient-derived cells that conserve patient-specific features has constituted a substantial progress in the study of PD, considering the great complexity and individual variability of the disease that encompasses unknown genetic and epigenetic factors, including aging, as well as environmental insults, has been recapitulated.

The use of patient-derived neural stem cell models has stirred up the field of PD research. Some works on PD have been done directly studying these stem cells (Sanberg, 2007) and some others by differentiating them into neural precursors or mature neurons (Le Grand et al., 2015; Yang et al., 2017). For instance, neural precursors as neurosphere models (free-floating clusters of neural stem cells) are widely used for the study of neuronal differentiation and neuronal disease (Matigian et al., 2010). For the study of mature-derived neurons, the development of induced pluripotent stem cells (iPSCs) has spawned a new approach to model PD allowing researchers to generate disease-specific DAn *in vitro* by reprograming somatic cells from patients with the disease (Fernandez-Santiago and Ezquerra, 2016). It is expected that the access to iPSCs-derived neurons from PD patients will shed light into mechanistic insights of PD pathogenesis and serves as a platform for drug screening and early diagnosis.

One of the latest applications of iPSC is the generation of different brain cell lineages to create brain organoids that resemble neuronal architecture, self-organization and cell to cell interaction from the physiologic brain. They are three-dimensional (3D) *in vitro* culture systems that recapitulate the developmental processes and organization of the developing human brain. These “mini-brains” provide a physiologically relevant model for the study of neurological development and disease processes that are unique to the human nervous system, together with other 2D and 3D models including neurospheres, neural aggregates, neural rosettes, and cortical spheroids. They all are emerging and promising models for the study of brain fragility and neurodegenerative diseases that will bring light into PD field in the next coming years but that are currently handicapped because of their novelty and setting up troubleshooting (Schwamborn, 2018).

Additionally, and despite being an exciting prospect for PD research, stem cell-derived neural lines and iPSC technologies have some other important inconveniences, including a considerable phenotypic variability unrelated to their genotype and their high maintenance costs and time-inefficiency (Jacobs, 2014). Additionally, stem cell or iPSC differentiation into neurons generally leads to low yields of DAn generation (Jacobs, 2014) thus obtaining heterogeneous cell pools where undifferentiated and DAn-derived cell types coexist (Fernandez-Santiago et al., 2015). Of note, whilst stem cells and iPSCs reprogramed from somatic cells highly rely on glycolytic metabolism, neurons are mainly energetically sustained by mitochondrial oxidative metabolism. In this scenario, one of the limitations of these cell models is the analysis of certain cell processes such as bioenergetics, oxidative stress or autophagy may become biased due to this confounding factor. Additionally, the genetic manipulation required to generate iPSC-derived DAn is frequently associated to genetic aberrations (e.g., copy number variations, somatic coding mutations, and chromosomal defects). Thus, the development of alternative patient-derived cell models is gaining in strength.

As previously mentioned, accumulating evidences suggest that PD is a multisystem disorder rather than a solely dopaminergic motor syndrome that encompasses central and peripheral clinical features (Djaldetti et al., 2009; Cersosimo and Benarroch, 2012). In line with this, PD pathological and molecular changes are also not confined in the CNS but are also present in the PNS and the organs that the latter innervates (Djaldetti et al., 2009). For instance, a great number of studies have reported α-synuclein deposits in many different peripheral tissues derived from PD patients (Tolosa and Vilas, 2015). On the other hand, many other alterations at molecular level including transcriptional changes, mitochondrial dysfunction and associated increased oxidative stress as well as autophagy deregulation have been described in PD-derived peripheral tissues such as muscle (Cardellach et al., 1993), blood cells including platelets and leukocytes (Haas et al., 1995; Muftuoglu et al., 2004; Mutez et al., 2014) and fibroblasts (Mortiboys et al., 2010; González-Casacuberta et al., 2018; Juarez-Flores et al., 2018), supporting the use of novel peripheral approaches.

The use of patient skin-derived fibroblasts has been widely utilized to model numerous diseases of metabolic, neurodegenerative, and lysosomal origin (Solini et al., 2004; McNeill et al., 2014; Alvarez-Mora et al., 2017; Konrad et al., 2017). For mitochondrial diseases, fibroblasts constitute the model of choice to diagnose and often to support research of these entities (Cameron et al., 2004; Soiferman and Saada, 2015; Ferrer-Cortès et al., 2016). Several studies in PD have used human skin-derived fibroblasts to investigate the molecular mechanisms underlying disease etiopathogenesis (Auburger et al., 2012). Skin-derived fibroblasts offer considerable advantages (Table 2): they constitute a patient-specific cellular system that retains the genetic (mutations, polymorphisms, polygenic risk factors, etc.) and epigenetic background of the patients while potentially preserving the specific environmental, toxic and cumulative age history. They show relevant expression of most PD genes which make them also suitable for the study of monogenic forms of PD (Auburger et al., 2012; Ivanov et al., 2016). Since they are accessible peripheral cells, they can be obtained from PD patients and healthy controls through an easy and minimally invasive procedure. Furthermore, fibroblasts can be propagated in culture, frozen, and stored for long periods of time, and transformed in cell types that exhibit molecular characteristics of the target tissue of the disease. It should be noted that fibroblasts make dynamic cell-to-cell contacts when cultured, which is similar to neuronal cells (Konrad et al., 2017).

**TABLE 2:** Advantages and disadvantages of using skin-derived fibroblasts as a cell model to study PD.

**Advantages**	**Disadvantages**
• Low facility and maintenance costs • Easy to obtain, propagate, maintain and store • Maintain genetic and epigenetic background of the patients • Reflect environmental cues and age-related events of patients • Relative poor invasiveness of the procedure • Presents relevant expression of most PARK genes • As primary cells, do not exhibit maximal glycolisis • Can be genetically manipulated to be reprogrammed to iPSCs and redifferentiated to dopaminergic neurons or to perform gene silencing or protein rescuing experiments	• As mitotic cells exhibit high renewal rates in contrast to the postmitotic nature of neural tissue • Not part of the central nervous system • Mixed metabolism (glycolitic and oxidative) to obtain cell energy • Mycoplasma contaminations are frequent and may lead to artificial phenotypes • Pure fibroblast culture is not achieved until passage 3 • Growing fibroblasts can be at different cell cycle phases representing a confounding factor

*Modified from Auburger et al. (2012).*

Studies in skin-derived fibroblasts from patients with sPD have thrown relevant and consistent information regarding molecular pathways altered in this type of neurodegeneration. A recent study showed that fibroblasts from sPD patients have higher growth rates, altered morphology, increased mitochondrial susceptibility to UV-exerted stress and autophagic alterations (Teves et al., 2017).

Other works focused on the study of molecular alterations in monogenic-PD patient fibroblasts have reported disease-relevant changes further supporting the adequacy of this model to study these forms of the disease. These changes include altered transcript (González-Casacuberta et al., 2018) and protein expression (Lippolis et al., 2015; Azkona et al., 2016), *SNCA* gene expression up-regulation (Hoepken et al., 2008), altered GCase enzyme activity (McNeill et al., 2014), microtubule destabilization (Cartelli et al., 2012), impaired autophagy (Dehay et al., 2012; Rakovic et al., 2013; Juarez-Flores et al., 2018), increased sensitivity to neurotoxins (Yakhine-Diop et al., 2014), bioenergetic deficits (Papkovskaia et al., 2012; Ambrosi et al., 2014; Juarez-Flores et al., 2018), mitochondrial alterations (Mortiboys et al., 2008, 2010), and enhanced apoptosis (Klinkenberg et al., 2010; Romani-Aumedes et al., 2014).

In summary, skin-derived fibroblasts show certain disadvantages (see Table 2) but constitute, at the same time, a patient-specific cellular system that, without genetic manipulation, can potentially recapitulate the main features of the disease (Auburger et al., 2012). In fact, many of the molecular hallmarks occurring in nigral DAn have been reported in fibroblasts from patients with sporadic and monogenic forms of the disease (Hoepken et al., 2008; Mortiboys et al., 2010; Ambrosi et al., 2014; Haylett et al., 2016).

## Molecular Mechanisms Underlying Pd Pathogenesis: Mitochondrial Dysfunction, Oxidative Stress and Autophagy Impairment

Most of accumulated evidences derived from studying brains of PD patients, animal or cell models of disease stand for common molecular mechanisms underlying PD pathogenesis. Among them, neuroinflammation, apoptosis, proteasomal dysfunction, and especially mitochondrial impairment, reactive oxygen species production (ROS) and autophagic failure, emerge as key players in PD development. However, there are different schools of thought regarding the triggers of the disease. Two main hypotheses raised: according to the “protein depot cascade hypothesis,” alpha-synuclein and other misfolded proteins stored as protein depots in neurons are the cause of PD. Other stand for the “mitochondrial cascade hypothesis,” that foresees the origin of PD in a defect in the oxidative phosphorylation (OXPHOS) system. Interestingly, protein deposition and bioenergetics appear to be closely related. Thus, alpha-synuclein can reduce OXPHOS function and OXPHOS deficiency can increase alpha-synuclein production (Cardoso, 2011; Haelterman et al., 2014).

In line with the “mitochondrial cascade hypothesis,” some primary mitochondrial diseases caused by monogenic mutations in mitochondrial-related genes that usually translate to OXPHOS deficiencies, clinically manifests as brain disease such as neurodegeneration and parkinsonism (Diez et al., 2017; Suomalainen and Battersby, 2017). In fact, mitochondria contribute to neurodegeneration through deficiencies in the mitochondrial respiratory chain (MRC) or OXPHOS and associated overproduction of ROS, as well as the accumulation of mitochondrial DNA (mtDNA) mutations or defects in its quality (deletions) or quantity (depletion) (Suomalainen and Battersby, 2017). Many evidences point out that mitochondrial dysfunction-derived oxidative stress is mainly centralized at the level of the MRC complexes I and III and play a central role in brain damage of PD patients (Poewe et al., 2017) (Figure 1).

The study of mitochondrial contribution to PD and other neurodegenerative diseases has been widely validated by the use of cybrids (Ghosh et al., 1999; Llobet et al., 2013). They allow the exploitation of mutation-independent, mitochondrial-derived impairments (age-associated, mostly). Arduíno et al. described the generation of cytoplasmic hybrid cells (or cybrids) as a promising cellular model for the study of sPD. This approach consists on the fusion of platelets harboring mtDNA from sPD patients with cells in which the endogenous mtDNA has been depleted (Rho0 cells). This allows the comparison of different mtDNAs in the same nuclear context. The sPD cybrid model has been successful in recapitulating most of the hallmarks of sPD (including CI dysfunction, ROS generation, loss of calcium homeostasis, changes in mitochondrial morphology, increased proton leak and decreased maximal respiratory capacity, as well as protein aggregation in the form of Lewy body-like inclusions) (Swerdlow et al., 1996; Cassarino et al., 1997; Sheehan et al., 1997; Ghosh et al., 1999; Trimmer et al., 2004; Esteves et al., 2009, 2010a,b; Arduíno et al., 2012, 2013; Llobet et al., 2013), thus constituting a validated model for addressing the link between mitochondrial dysfunction and sPD pathology.

Mitochondrial function is essential in almost all cells of the organism, but specially in neurons. There are different reasons to explain their dependence on proper mitochondrial function, controlled oxidative stress production and adequate mitochondrial replacement through autophagy.

Neural metabolism is highly oxidative. Glucose is the obligatory energy substrate of the adult brain. However, under certain circumstances the brain has the capacity to use alternative blood-derived energy substrates, such as ketone bodies (during starvation or development) (Magistretti, 2006) and lactate (during periods of intense physical activity) (van Hall et al., 2009). Once inside the cell, glucose can be metabolized through glycolysis (leading to lactate production or mitochondrial metabolism) or through the pentose phosphate pathway (as glycogenesis can only be performed in astrocytes). Both mitochondrial metabolism and pentose phosphate pathway are the proper bioenergetic pathways enhanced in physiologic conditions to provide of ATP and antioxidant power to the cell, respectively. Contrarily, the metabolic activation of lactate production through the anaerobic glycolysis may be detrimental for long-term neuronal function and should be only sustained in certain punctual circumstances (Falkowska et al., 2015).

Metabolic oxidative activity of neurons explains they dependence on mitochondria to obtain energy. Noticeably, brain is one of the highest energy-demanding organs of the body due to the its intrinsic physiological activity. It is constituted by postmitotic cells (neurons) with less capacity for cellular regeneration compared to other organs. Thus, it is believed to be particularly vulnerable to mitochondrial dysfunction, ROS damaging effects and detrimental autophagic renewal of cell components, that may explain why is prone to manifest clinical evidences of mitochondrial, oxidative or autophagic alterations (Picard and McEwen, 2014). This is especially relevant in the case of DAn that synthesize the most pro-oxidant neurotransmitter of the CNS (DA), thus becoming especially vulnerable to oxidative environments, mitochondrial failure or autophagic imbalance.

In this scenario, mitochondrial dysfunction, associated oxidative stress and autophagic development becomes critical for neuronal survival. Several lines of evidence have implicated mitochondrial dysfunction as a key element in PD pathogenesis. Reduction of mitochondrial CI activity has been reported in several tissues isolated from PD patients including the CNS (Schapira and Gegg, 2011). In addition, the target genes of the mitochondrial master transcriptional regulator PGC1α have been reported to be under expressed in PD, together with strong evidences of increased oxidative stress and reduced autophagic function that may be finally responsible for defective mitochondrial and protein depot (Schapira and Gegg, 2011; Nixon, 2013; Siddiqui et al., 2015).

Unfortunately, when postmortem brain tissue of PD patients is studied, most of DAn have disappeared (Teves et al., 2017), thus difficulting the establishment of any potential etiologic causal link. In consequence, the study of experimental models of PD conveys the opportunity to foresee the triggers of neuronal degeneration.

As previously mentioned, initial models of PD were developed by using MPTP, a mitochondrial neurotoxin that specifically targeted DAn in primates and mice which was discovered to produce parkinsonism in humans (Langston, 2017). MPTP-based models and many other models using mitochondrial neurotoxins such as 6-hydroxydopamine (6-OHDA), rotenone or paraquat, have been used over the past years in the PD research field to replicate features of disease neuropathology (Jagmag et al., 2015). On the other hand, the depletion of mitochondrial proteins in mice that are essential for mtDNA maintenance (TFAM and En1) leads to neuronal degeneration of DAn in the SN which accounts for the development of several important features of PD neuropathology (Pickrell et al., 2013).

In PD patients, reduction of mitochondrial CI activity has been reported in several isolated tissues including the SN and peripheral tissues (Schapira and Gegg, 2011). In addition, the downregulation of the target genes of the master transcriptional regulator involved in mitochondrial biogenesis (peroxisome proliferator-activated receptor gamma coactivator 1-alpha or PGC-1α) have been reported in PD (Siddiqui et al., 2015). Low levels of α-synuclein have been encountered in mitochondria in physiologic conditions and *in vitro* abnormal accumulation of this protein has proven to lead to mitochondrial CI dysfunction and associated oxidative stress, importantly linking these two events that have been repeatedly reported in PD (Poewe et al., 2017).

Different levels of ROS damage have been reported within the target brain region that undergoes selective neurodegeneration in PD. Specifically, lipid peroxidation markers such as 4-hydroxynonenal and malondialdehyde, have been identified in the SN of PD patients (Dias et al., 2013). However, it remains elusive whether oxidative stress occurs early in the disease or later during the decease of neurons and thus, as a consequence of cell degeneration.

Mitochondria are not isolated or static entities but instead are highly dynamic organelles that are transported on cytoskeletal proteins responsible for mitochondrial trafficking and are continuously subjected to fusion and fission processes in order to maintain their homeostasis (Johri and Beal, 2012). Mitochondrial dynamics have also been reported to be altered in PD (Van Laar and Berman, 2009). Similarly, mitochondrial turnover and quality control through selective autophagy (mitophagy), or mitochondrial relationship with other organelles as endoplasmic reticulum and its associated membranes (MAMs) have also been associated to PD (Hattori et al., 2017). Mitochondrial dysfunction and oxidative stress are associated with the impairment of the autophagy process through the accumulation of damaged mitochondria due to their defective turnover and through depletion of lysosomes, evidencing that different molecular pathways involved in PD pathogenesis are intimately related (Poewe et al., 2017).

Although our understanding of the regulatory pathways that control autophagy is still limited, recent advances have shed light on the importance of autophagy in a panoply of physiological processes and human diseases including neurodegeneration (Jing and Lim, 2012). The post-mitotic status of neurons prone them to be strictly dependent on optimal regulation of autophagy for the removal of dysfunctional or obsolete cell constituents, especially as the brain ages. In fact, the most prevalent pathological feature of many neurodegenerative diseases is the aggregation of misfolded proteins and the loss of certain neuronal populations (Guo et al., 2018), closely related to lysosomal function. This becomes more evident with the observation that the brain is often the most severely affected organ in primary lysosomal disorders and that mutations in genes involved in autophagy are causatively linked mechanisms to neurodegenerative disorders with exceptional frequency. This link between lysosomal performance and neurodegenerative diseases explains the high prevalence of genetic lysosomal variants that are found in PD by genome wide association studies (Levine and Kroemer, 2019).

On the other hand, it has been reported that in neurodegenerative disorders, such as Alzheimer’s disease, amyotrophic lateral sclerosis and familial PD, defects arise at different stages of the autophagy pathway and have different implications for pathogenesis and therapy (Nixon, 2013). Recent research in the field of PD points out to alterations in specific steps of autophagic processes that could be of high relevance in the etiopathogenesis of the disease (Jing and Lim, 2012; Nixon, 2013; Guo et al., 2018) (Figure 1). For instance, an upregulation of macroautophagy following an overwhelmed chaperone-mediated autophagy (CMA) system as a result of overexpression of misfolded aggregates of α-synuclein has been shown in mice and *in vitro* PD model systems (Spencer et al., 2009; Yu et al., 2009; Ebrahimi-Fakhari et al., 2011). Moreover, an accumulation of autophagosomes (AP) and an early decrease in lysosome content as a result of lysosomal membrane destabilization and cytosolic release of cathepsins has been reported in DAn of a neurotoxin-based mouse model (Dehay et al., 2010). In addition, a decrease in lysosomal acidification and consequent decline of lysosomal protein turnover has been reported in mice overexpressing α-synuclein (Stefanis et al., 2001; Cuervo et al., 2004).

In *post mortem* brain samples of PD patients dysfunctional lysosomes and accumulation of AP were observed in neurons, indicating a pathogenic role of autophagy in PD (Guo et al., 2018).

### Mitochondrial Dysfunction, Oxidative Stress, and Autophagy Disruption in Familial PD

**FIGURE 2:**
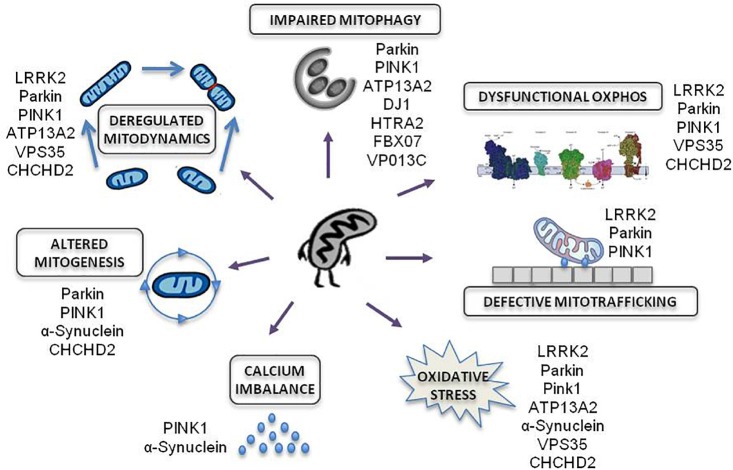
Mitochondrial effects of genetic mutations associated to familial PD. Modified from Park et al. (2018).

Amongst all familial forms of PD, late and early onset PD associated to mutations in *LRRK2* and *PRKN* genes, respectively, are responsible for the most frequent dominant and recessive inherited forms of PD. These genes have emerged as promising examples of disease due to their established role in commanding bioenergetic and autophagic balance (Figure 2).

Numerous genes responsible of inherited PD cause the impairment of essential functions for mitochondrial homeostasis: OXPHOS function, mitochondrial trafficking, oxidative stress, calcium imbalance, mitochondrial biogenesis, mitochondria dynamics and mitochondrial autophagy.

#### *LRRK2*-Associated PD (LRRK2-PD)

The *LRRK2* gene (at the PARK8 *locus*) is located in chromosome 12, contains 51 exons and spans a genomic distance of 144 kb that includes 7500 nucleotides of coding sequence. Up to date, about 80 “probably pathogenic” and seven pathogenic *LRRK2* mutations have been described, being the G2019S (G2019S-*LRRK2*) the most frequent pathogenic mutation (Corti et al., 2011). Most of *LRRK2* mutations correspond to missense variants, which, along with the dominant inheritance, are consistent with a gain-of-function pathogenic mechanism (Corti et al., 2011). A number of non-pathologic variants are also known and some others variants that may act as PD risk factors have been reported using genome wide association studies (Islam and Moore, 2017).

**FIGURE 3:**
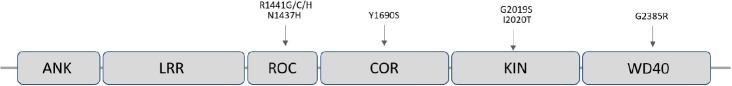
Schematic representation of the LRRK2 protein showing its functional domains and the most frequent pathogenic missense point mutations responsible for PD.

*LRRK2* encodes a 2527 amino-acid multi-domain protein (LRRK2), which is also known as dardarin, from the Basque word “dardara” which means trembling. *LRRK2* has the particular feature of encoding a leucine-rich repeat (LRR), a ROC-COR GTPase, a mitogen-activated protein kinase, and WD40 domains in the same protein (Singh et al., 2019) (Figure 3). Of note, pathogenic mutations appear to be located in functionally relevant regions of the protein such as in the specific case of the G2019S-*LRRK2* mutations that affect the kinase domain making it more active (Corti et al., 2011) (Figure 3). LRRK2 is expressed in most organs including brain, heart, liver, and circulating immune cells.

Mutations in *LRRK2* account for ∼10% of familial PD and for a significant fraction of sPD cases (Kalinderi et al., 2007). *LRRK2* mutation frequencies vary between ethnic groups, being North African Arabs and Ashkenazi Jews the most affected populations (Correia Guedes et al., 2010). The G2019S-*LRRK2* mutation is responsible for 1% of apparent sPD and 4% of familial PD worldwide (Healy et al., 2008). Importantly, even when proven pathogenic mutations are present, penetrance is age dependent and estimated between 30 and 74% (Ozelius et al., 2006). This fact makes the *LRRK2*-mutation carriers a very interesting target of study, as they present a subclinical stage with molecular alterations potentially determinant for disease progression.

Clinically, *LRRK2*-associated PD (*LRRK2*-PD) presents with a PD-typical phenotype with no sex association. A large systematic review of *LRRK2*-PD case reports that age of onset is around 57 years, with a mean disease duration of 10 years. The cardinal PD symptoms were reported with the following frequency: bradykinesia in 99%, rigidity in 99%, tremor in 88% and postural instability in 65%, while atypical signs of PD have been reported only anecdotally. In addition, autopsies of such patients showed prominent loss of melanized DAn in the SNpc (Trinh et al., 2018). Disease progression is slow and response to treatment is as good as in sPD (Corti et al., 2011). *LRRK2*-PD has demonstrated an unprecedentedly significant role of LRRK2 in PD pathogenesis as most of the clinical and pathological features are indistinguishable from those of sPD (Gosal et al., 2005). Thus, since its discovery, great efforts have been focused on the study of this form of the disease.

Within cells, *LRRK2* associates with various intracellular membranes and vesicular structures including the endosomes, the lysosomes, the multivesicular bodies, the outer mitochondrial membrane (OMM), lipid rafts, microtubule associates vesicles, Golgi complex, and the endoplasmic reticulum (Cookson, 2012), thus it is highly associated to MAMs. Accordingly to its multi-domain nature, LRRK2 has been implied in many cellular functions such as cytoskeleton remodeling, vesicle trafficking and movement, protein translation, autophagy and mitochondrial function homeostasis (Cookson, 2010; Liu et al., 2012; Taymans et al., 2015; Roosen and Cookson, 2016; Juarez-Flores et al., 2018; Price et al., 2018). Specifically, studies consisting of modifications in the expression of *LRRK2* in different neural-cell based models have reported an altered synaptic vesicle trafficking and endocytosis (Shin et al., 2008; Piccoli et al., 2011). LRRK2 dysfunction has also been demonstrated to play a role in different pathologic scenarios, such as ∝-synuclein phosphorylation, microtubule dynamics, alterations in the uncoupling protein system (UPS) as well as in neurite growth and branching regulation that may trigger neurodegeneration (Dächsel et al., 2010). Silencing of LRRK2 reduced the inflammatory response in different human cell-derived and animal models (Lopez de Maturana et al., 2014).

A growing body of evidence supports a role for LRRK2 in mitochondrial dynamics and function. In fact, it interacts with a number of crucial proteins that regulate mitochondrial dynamics such as dynamin-related protein1 (DRP1), Mitofusin (MFN1) and 2 and optic atrophy1 (OPA1) (Wang et al., 2012; Stafa et al., 2014; Park et al., 2018). Thus, *LRRK2* might directly affect mitochondrial homeostasis while indirectly regulating it through autophagy and cytoskeletal dynamics (Singh et al., 2019). This hypothesis is also supported by many studies reporting mitochondrial dysfunction in various animal models of G2019S-*LRRK2* PD, in *postmortem* human tissues from LRRK2-PD patients (Mortiboys et al., 2010; Cooper et al., 2012; Sanders et al., 2014; Yue et al., 2015) and in different patient-derived cell models (Niu et al., 2012; Wang et al., 2012; Cherra et al., 2013; Su and Qi, 2013). Such studies reported mtDNA damage, decreased mitochondrial membrane potential (MMP) and ATP production, as well as altered mitochondrial dynamics and mitophagy (Singh et al., 2019). In addition, a protective role against oxidative stress has been reported for wild-type LRRK2, which seems to be lost in mutant forms of the protein (Liou et al., 2008).

#### Studies in Fibroblasts From *LRRK2*-Associated PD

Mitochondrial phenotypes have been characterized in *LRRK2*-fibroblasts at baseline and under conditions of pharmacological stress. The most common pharmacological approaches used to date include mitochondrial toxins such as MPTP, valinomycin, oligomycin, CCCP, and rotenone. As these approaches may mimic mitochondrial toxicity, they are far from dissecting the mitochondrial pathways affected under physiological conditions (Trentadue et al., 2012). Smith et al. (2015) demonstrated an increased sensitivity to valinomycin in a subset, but not all, of fibroblasts derived from PD patients, pinpointing again the great interindividual variability of the disease and, outstandingly, that the molecular characteristics of patient-derived cell models do not always correlate with the clinical presentation of the disease.

Mortiboys et al. (2010) reported for the first time mitochondrial alterations in fibroblasts of human G2019S-*LRRK2* mutation carriers, consisting of reduced MMP, reduced intracellular ATP levels, mitochondrial elongation and increased mitochondrial interconnectivity. These findings were further confirmed by Papkovskaia et al. (2012), who described decreased MMP and ATP levels as well as increased proton leakage and ROS levels with the associated increase in uncoupling protein 2 (UCP2) in fibroblasts from G2019S-*LRRK2* PD patients. Several studies have repeatedly observed alterations in mitochondrial dynamics such as increased mitochondrial fragmentation in fibroblasts from *LRRK2*-PD patients (Su and Qi, 2013; Grünewald et al., 2014; Smith et al., 2015; Falkenburger et al., 2016).

A recent study compared mitochondrial function and autophagy in fibroblasts of G2019S-LRRK2-mutation carriers without clinical symptoms (so called non-manifesting carriers or NMC), with patients harboring G2019S-LRRK2-mutation and clinical manifested PD. Interestingly, fibroblasts of NMC showed an enhanced mitochondrial performance upon forcing mitochondrial oxidative metabolism with galactose and upregulation of autophagy (Juarez-Flores et al., 2018). These findings suggested that the exhaustion of the bioenergetic and autophagy reserve might contribute to the onset of clinical PD symptoms. Other authors have reported heightened autophagic flux and higher expression of autophagy markers as well as an increased mitophagy in G2019S-LRRK2-mutation carriers with clinical diagnosed PD (Smith et al., 2015; Su et al., 2015). The reduction in mitophagy and increased ROS production has been associated to defective histone acetyltransferase and deacetylase activities contributing to cell death also in LRRK2-fibroblasts (Yakhine-Diop et al., 2019). Additionally, the novel role of key regulators of autophagy (as TMEM230) interacting with Rab proteins as Raba or Rab32been described in LRRK2-patients fibroblasts is emerging as a promising new target in disease (Waschbüsch et al., 2014; Kim et al., 2017).

#### *PRKN*-Associated PD (*PRKN*-PD)

Other forms of PD have also been genetically associated to mitochondrial and autophagic imbalance, in this case through a recessive inheritance. This is the case of PRKN.

**FIGURE 4:**
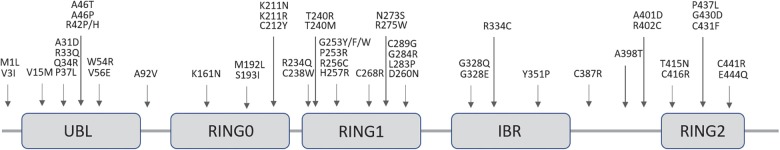
Schematic representation of the Parkin protein showing its functional domains and the most frequent pathogenic missense point mutations responsible for PD. Dozens of alternative frameshift and nonsense mutations, insertions, deletions, duplications or triplications have also been associated to disease.

The locus of *PRKN* is mapped to the telomeric region of the long-arm of chromosome 6. More than 170 different mutations have been identified throughout the sequence of this particularly large gene (1.35 Mb) ranging from point and missense mutations to large deletions or multiplications and small deletions/insertions (Bruggemann and Klein, 1993; Klein and Westenberger, 2012) (Figure 4). Rare deletions extending in the neighboring *PRKN* coregulated gene (*PACRG*) result in the same early onset parkinsonism phenotype (Corti et al., 2011).

PRKN is a 465 amino acid protein that contains an NH2-terminal homologous to a ubiquitin-like domain (UBL) followed by three really interesting new gene (RING) finger domains (RING 0–2) separated by an In-Between-RING (IBR) domain in the COOH-terminal part, each of which bind two Zn^2+^ (Zhang et al., 2015) (Figure 4). Functionally, PRKN is a member of a family of E3 ubiquitin protein-ligases responsible for the labeling of selected cargos, such as obsolete proteins and organelles, which need to be degraded through the ubiquitination process. This process comprises the transfer of activated ubiquitin molecules to the lysine residues of specific substrate proteins. Depending on the site and type of ubiquitination (mono, poly or multi-ubiquitination), certain cell signaling processes are activated, including proteosomal degradation but also non-degradative signaling roles (Dawson and Dawson, 2010).

Along with the original discovery of the PRKN function as an E3 ubiquitin ligase in PD-associated *PRKN* mutations, the hypothesis that loss of PRKN function would lead to the toxic accumulation of one or several of its substrates raised. To date, no less than 25 PRKN putative substrates have been reported and new substrates continue to emerge periodically, especially those related to mitochondria (Zhang et al., 2015). In addition, many dynamically regulated ubiquitination sites in dozens of proteins have been identified, with strong enrichment for OMM proteins, indicating that PRKN dramatically alters the ubiquitination status of the mitochondrial proteome (Sarraf et al., 2013).

Nigral cell loss in *PRKN*-PD patients appears to be caused by a loss of function of the protein due to biallelic homozygous or compound heterozygous mutations in the *PRKN* gene. However, there is an ongoing debate with regard to whether heterozygous *PRKN* mutations may confer increased susceptibility to PD as heterozygous *PRKN* pathogenic variants have been detected in a large number of individuals with PD (Bruggemann and Klein, 1993; Mortiboys et al., 2008).

Although the population-based prevalence of *PRKN*-PD is largely unknown (Bruggemann and Klein, 1993), it is thought that *PRKN* mutations account for up to 50% of recessive familial forms and 80% in those patients with a PD onset before the age of 20 years (Corti et al., 2011). Women and men are equally affected, with an age at onset usually <40–50 years (Mizuno et al., 2001) although some individuals may not develop PD until age 60 or 70 years (Klein et al., 2000; Lohmann et al., 2003). In addition to an earlier age at onset, *PRKN-*PD patients show a clinical phenotype similar to that of sPD being bradykinesia and tremor amongst the most common signs, but also a number of specific clinical features. *PRKN*-PD is also characterized by a relatively benign course with slow progression, remarkable and maintained response to low levodopa doses but with frequent severe treatment-related motor complications such as early motor fluctuations and the development of dyskinesias (Cheon et al., 2012). Pyramidal signs, cerebellar features, and psychiatric disorders have been reported, but dementia or dysautonomia seem to be rare (Corti et al., 2011; Johansen et al., 2018).

In the limited neurophathologic studies, *PRKN* mutations are associated with selective DAn loss in the SN and some cases reported a moderate decrease of noradrenergic neurons in the *Locus coeruleus* with gliosis and without LB. However, a few cases of LP have been reported in PRKN-PD, especially those associated to a later onset of the disease (Bruggemann and Klein, 1993; Johansen et al., 2018).

One of the best characterized functions of PRKN is its role in the process of mitophagy, which is the selective targeting of a damaged mitochondrion for autophagy. Compelling evidence suggests that PRKN acts together with and downstream of PINK1 in a common mitochondrial quality control pathway responsible for the detection and clearance of damaged mitochondria through mitophagy (Eiyama and Okamoto, 2015). In healthy mitochondria, PINK1 is constitutively imported into the OMM and inner mitochondrial membranes (IMM), cleaved by several proteases and subsequently degraded. Loss of MMP impedes the import of PINK1 in the IMM, thereby stabilizing PINK1 on the OMM and consequently recruiting PRKN from the cytosol. In its native state, PRKN is auto-inhibited by its N-terminal UBL domain, which blocks the binding site for any incoming E2 ubiquitin conjugate, required for PRKN ubiquitination activity. Upon mitochondrial depolarization, PINK1 phosphorylates cytoplasmic PRKN in its UBL domain, relieving PRKN autoinhibition (Eiyama and Okamoto, 2015). Activated PRKN ubiquitinates many OMM proteins including VDAC1, mitofusins and the translocase of the OMM 20 (TOMM20) (Kondapalli et al., 2012; Ivankovic et al., 2016). Together, PINK1 and phosphorylated PRKN extensively modify the OMM with phosphorylated ubiquitin (pUb) chains. pUb chains serve as a mitochondrial receptor for further allosteric activation and recruitment of PRKN to the OMM, resulting in a self-amplifying feed-forward loop. Ubiquitination of these substrates primes mitochondria for recruitment to phagophores that then mature to AP and fuse with lysosomes resulting in the degradation of dysfunctional mitochondria (Ichimura et al., 2013). In addition, recent evidence suggests that PRKN is also involved in the aggresome-autophagy pathway in which PRKN promotes the sequestration of misfolded proteins into aggresomes and its subsequent clearance by autophagy (Olzmann and Chin, 2008; Yung et al., 2016).

On the other hand, PRKN has been implicated in mitochondrial biogenesis specifically, through the regulation by ubiquitination of the protein levels of one of its substrates named PARIS (ZNF746) (Shin et al., 2011). PARIS represses the expression of the transcriptional coactivator PGC-1α, which is considered a master regulator of mitochondrial biogenesis. In this line, PARIS has been reported to accumulate in models of PRKN inactivation and in human PD brain (Shin et al., 2011). Thus, PRKN potentially acts as an intermediary between mitochondrial biogenesis and autophagy, by both blocking mitochondrial biogenesis and mitochondrial turn-over, thus resulting in mitochondrial aging.

#### Studies in Fibroblasts From *PRKN*-PD Patients

Amongst all the studies using skin-derived fibroblasts as a cell model for PRKN-PD, it is worth stressing that many of them have focused on studying mitochondrial function leading to controversial outcomes.

Alterations in the enzymatic activities of the MRC have been previously reported in PRKN-PD fibroblasts (Mortiboys et al., 2008; Grunewald et al., 2010; Pacelli et al., 2011). Mortiboys et al. (2008) and Pacelli et al. (2011) described CI enzymatic deficiency in *PRKN*-PD fibroblasts, while Grunewald et al. (2010) observed preserved enzymatic activities in isolated mitochondria from a larger cohort (Mortiboys et al., 2008; Grunewald et al., 2010; Pacelli et al., 2011). Mitochondrial complex IV deficiency has only been described by Pacelli et al. (2011) in two PRKN-PD fibroblasts lines while others have reported unaltered enzymatic activity of this complex (Mortiboys et al., 2008; Grunewald et al., 2010). Mitochondrial respiration is frequently measured to assess how MRC enzymatic activities translate to global mitochondrial function. Haylett et al. (2016) and Zanellati et al. (2015) consistently observed increased basal mitochondrial respiration in PRKN-PD fibroblasts but reported opposite outcomes in ATP-linked respiration. In contrast to these findings, a previous study described an overall decrease in all respiratory parameters of PRKN-PD fibroblasts (Pacelli et al., 2011).

Mitochondrial membrane potential has also been widely explored as a general marker of mitochondrial integrity in PRKN-PD fibroblasts. While two authors were not able to demonstrate alterations in this parameter (Grunewald et al., 2010; Haylett et al., 2016), others reported decreased MMP (Zanellati et al., 2015; Koentjoro et al., 2017), especially when exposing cells to mitochondrial-challenging conditions (Mortiboys et al., 2008; Grunewald et al., 2010).

Many evidences point to the involvement of PRKN in the entire process of mitochondrial dynamics, including organelle biogenesis, fusion/fission, and mitochondrial clearance via mitophagy (Lim et al., 2012). In this context, mitochondrial network morphology has been of interest in *PRKN*-PD fibroblasts studies but with controversial results (Mortiboys et al., 2008; Grunewald et al., 2010; Pacelli et al., 2011; van der Merwe et al., 2014; Zanellati et al., 2015; Haylett et al., 2016). Although some studies seem to agree that mitochondrial length is conserved in these cells (Mortiboys et al., 2008; van der Merwe et al., 2014; Haylett et al., 2016), others have observed a fragmented mitochondrial network (Pacelli et al., 2011; Zanellati et al., 2015). While most of studies did not show significant alterations in mitochondrial branching (Grunewald et al., 2010; van der Merwe et al., 2014; Zanellati et al., 2015), Haylett et al. (2016) observed decreased levels and Mortiboys et al. (2008) reported increased rates. In line with this, mitochondrial content has been assessed in several works but only Grunewald et al. (2010) reported a significant increase in this feature in *PRKN*-PD fibroblasts whereas others have observed conserved (Mortiboys et al., 2008; van der Merwe et al., 2014) or decreased levels (Pacelli et al., 2011).

As previously discussed, oxidative stress is a hallmark of mitochondrial dysfunction that has often been related with neurodegeneration, specifically in PD (Guzman et al., 2010; Surmeier et al., 2011; Poewe et al., 2017). In accordance, previous authors demonstrated increased protein and lipid oxidation in small cohorts (Grunewald et al., 2010; Pacelli et al., 2011).

Surprisingly, to our knowledge, studies assessing mitophagy or autophagy in *PRKN*-PD fibroblasts are scarce. Only recently, Koentjoro et al. (2017) elegantly demonstrated that a *PRKN*-PD patient fibroblast cell line failed in initiating mitophagy upon induction of mitochondrial depolarization. Interestingly, they also examined an unusual homozygous *PRKN* mutation carrier who did not develop clinical PD by her eight decade and found preserved mitochondrial function due to the induction of a PINK1/Parkin-independent mitophagy mediated by Nix, which is a selective autophagic receptor located on the OMM (Koentjoro et al., 2017).

Other studies in *PRKN*-PD fibroblasts have reported alterations in alternative important cell processes which represent promising targets of disease pathogenesis to be further explored. For instance the regulation of endoplasmic reticulum-to-mitochondrial contacts by Parkin via Mfn-2 (Basso et al., 2018). Also, Pacelli et al. (2019) reported altered severe damping of the bioenergetic oscillatory patterns associated to circadian rhythms and molecular clockworks in fibroblasts from PRKN-PD patients that may conditioning mitochondrial quality control and mitophagy. One study performing a whole-genome expression analysis by RNA-sequencing found that different *PRKN* mutations were associated with a large number of gene expression changes at the transcriptome level (González-Casacuberta et al., 2018). Specifically, authors reported the upregulation of 1C-dependent anabolic biosynthetic pathways, which has been related with the activation of the mitochondrial integrated stress response (ISRmt) in front of mitochondrial dysfunction (Bao et al., 2016; Celardo et al., 2017; Suomalainen and Battersby, 2017). Additional studies in PRKN-PD fibroblasts have reported alterations in the protein expression and lipidome profiles (Lippolis et al., 2015; Lobasso et al., 2017) as well as cytoskeleton alterations such as microtubule destabilization (Cartelli et al., 2012; Vergara et al., 2015).

The characterization of fibroblasts of PD patients point out disrupted pathways to be targeted and therapeutic platforms, but some concerns and controversies arise. The low reproducibility of mitochondrial function analysis presented in most studies performed up to date could be attributed to the small sample sizes tested. Also differences in methodological approaches, protocols and experimental conditions (e.g., site of skin biopsy, passage number of cells, etc.) may partially account for the large variation obtained. For instance, the use of different high-resolution respirometry approaches in which oxygen consumption is measured from seeding fibroblasts or from cells in suspension. Similarly, assessing MRC enzymatic activities in intact cells or in mitochondrial enriched fractions may contribute to outcome disparities. Moreover, all the studies were performed in glycolytic conditions that may partially unveil mitochondrial deficits and contribute to controversy (Mortiboys et al., 2008; Grunewald et al., 2010; Pacelli et al., 2011; van der Merwe et al., 2014; Zanellati et al., 2015; Haylett et al., 2016). In this sense, the use of alternative sources of energy, as galactose, serves for two purposes: to force and challenge oxidative metabolism (usage widely extended for the diagnosis of mitochondrial disorders) and mimic neuronal metabolism (mainly based in OXPHOS function). Novel studies focused in exploring mitochondrial or autophagic function in galactose may be useful to unveil pathogenic mechanisms of disease.

In summary, the particular area of research focused on the study of mitochondrial function in *PRKN*-PD fibroblasts has proved to be contentious, with several groups either describing similar defects or no apparent abnormalities. It would be of great importance that researchers join efforts on homogenization of protocols and analyzing a more significant number of *PRKN*-PD patient-derived cells in order to unveil if mitochondrial and autophagic dysfunction is a crucial event in PRKN-PD pathogenesis.

## Disease Modifying Therapies

Since the disseverment of PD, different therapeutic options have been developed to ameliorate the symptoms of PD. The first one to be developed was levodopa, in 1960, a precursor that is transformed to DA in the brain, supplying the amount of DA that degenerated neurons are not able to produce. Other medications include DA agonists and monoamine oxidase-B (MAO-B) or Catechol-O-methyltransferase inhibitors (COMT) inhibitors, selegiline, and rasagiline, that decrease the activity of MAO-B, enzyme responsible of degrading DA. There are also other options to severe cases who do not respond to DA based on the use of apomorphine and duodopa administered by pumps, surgical interventions or deep brain stimulation (Rizek et al., 2016).

However, these treatments are supportive, only control the symptoms of the disease, the neurodegeneration is not stopped or reversed, consequently, there are no curative treatments for PD.

To unveil the pathophysiology of the diseases and develop new therapeutic strategies to reduce the impact of PD and find a cure is essential to develop novel models of disease.

The experimental models herein discussed hold potential for the development of PD modifying therapies. The complementary assay of any potential candidate in different experimental models confers strength to the potential therapeutic efficacy before translation into the clinical settings. Patient-derived cell models offer usefulness either as platforms for testing novel therapeutic approaches or for prompting the discovery of novel targets from disrupted pathways reported in these models.

The use of these experimental models in PD has permitted the discovery of different therapeutic candidates, with different degree of evidence on their potential therapeutic activity and security concerns. This is the case of FGF20, echinacoside, rosmarinic acid or autophagic modulators, as Threalose or Torin 1, among many others, targeting different disrupted cell pathways in disease (Liang et al., 2019; Lv et al., 2019; Wang et al., 2019). Depending on the subtype of PD, specific treatments have been proposed. This is the case of *LRRK2*-PD carriers, where the use of LRRK2 inhibitors has been proposed. They are currently being tested in clinical I trials. Unfortunately, their systemic action may unveil secondary effects, somehow bypassed by the targeting of specific neural effectors (as PAK6 or Rab GTPases) to modulate neural disrupted protein and organelle trafficking in PD (Kiral et al., 2018). Similarly, for *PRKN*-patients, selective mitochondrial drugs have been proposed. Experimental data supports the use of fusion or fission inhibitors (as MDIVI-1), that still rank in experimental settings (Manczak et al., 2019). Antioxidant and mitochondrial principles (as coenzyme Q, that failed in a phase III assay) and peroxisome proliferator-activated receptor-γ agonists that reduces proinflammatory cytokines and modulate mitochondrial biogenesis (as Pioglitazone) were also tested in clinical trials, but failed to demonstrate further efficacy.

Apart from symptomatic treatments (such as levodopa or surgical interventions), disease modification through neuroprotection remains as the main milestone in PD research. Neuroprotection tested by pramipexole (CALM-PD), ropinirole (REAL-PD), and pramipexol (PROUD-PD) failed to establish disease modification (Bartus et al., 2013; Obeso et al., 2017). In this sense, calcium channel blockers aimed to prevent calcium influx on nigral neurons are being tested (isradipine is being evaluated in phase I and II clinical trials), together with compounds able to increase urate antioxidant protection (inosine is undergoing phase II studies) (Obeso et al., 2017).

Additionally, aiming to support neuroprotection through the enhancement of neuronal viability, trophic factors are also being evaluated in PD, showing moderate or null therapeutic success. Of them, glial family ligands as glial derived neurotrophic factor (GDNF) and neurturin in preclinical studies demonstrated strong neuroprotection in multiple animal models. However, multiple clinical trials, including 2 phase II trials, failed to demonstrate their efficacy or showed significant side effects (Kordower et al., 2000; Obeso et al., 2017).

Alpha-synuclein has become lately the major target for PD therapeutics. Initial preclinical efforts concentrated on synuclein-lowering treatments such as siRNAs directed against alpha-synuclein, that resulted toxic in animals. Novel attempts focused to disaggregate aggregated synuclein, facilitate its clearance by augmenting autophagy pathways, or using antibodies to prevent its propagation from the periphery to the brain and once in the brain across the neural axis. Vaccines against alpha-synuclein as both active and passive immunization approaches have been attempted. Active immunotherapy attempts to stimulate the immune system against specific antigens (Bergström et al., 2016). Passive immunotherapy uses monoclonal antibodies against alpha-synuclein molecule. Initial phase 1 safety trials are currently underway and show promising results in terms of safety and tolerability profiles. The enhancement of glucocerebrosidase (GBA) lysosomal activity to reduce alpha-synuclein levels is also being tested through small molecule chaperones in clinical trials (McNeill et al., 2014).

Probably the next coming years will open future perspectives for the development of new supportive and curative therapies in PD, where personalized medicine, mainly based on genetic and molecular counseling, will help to direct specific PD patients to a wide panoply of therapies.

The development of novel therapeutic options will depend of the efficacy of candidate compounds previously tested in preclinical settings and experimental models of disease, as the herein described, and the target of disrupted pathways, as the herein explained.

## Conclusion

Parkinson’s disease encompasses a wide panoply of genetic and molecular etiologies leading to common clinical manifestations. Different schools of thought differ in considering either mitochondria or protein deposition-cascade as the triggers of PD, but all they convey that PD pathogenesis is associated to the deregulation of both mitochondrial and autophagic clearance pathways, supporting its role in the disease. Mitochondrial dysfunction and their turnover through autophagy directly targets some types of PD (as those carrying mitochondrial or autophagic mutations) but also stand at the base of the rest of PD by providing the overdose of energy needed to support alternative deregulated pathways while maintaining oxidative stress levels within control ranges. Thus, proper mitochondrial and autophagic function protects against PD and exhaustion of mitochondrial and autophagy contributes to PD development, independently of the genetic base. These molecular alterations have been consistently reported in skin-derived fibroblasts from PD patients carrying mutations in *LRRK2* and *PRKN* genes. These findings demonstrate the presence of molecular damage characteristic of the PD target tissue beyond the CNS and the usefulness of these patient-derived cells to model PD, models that can be metabolically upgraded to resemble neuron behavior and challenge mitochondrial and autophagic function by the use of galactose.

Current research gaps in PD research stand for the development of novel therapeutic candidates aimed to promote healthy brain aging and avoid or even cure PD, probably based in personalized-medicine guided by genetic and molecular counseling. New generation sequencing will increase the number of genes responsible of familial PD and the number of genetic risk factors accounting for sporadic PD, thus unveiling molecular imbalances underlying PD. Novel compounds against these targets will be discovered in experimental settings and disease models to set the path for further clinical trial testing. Complementary models of disease will be needed to dissect the disrupted pathways in PD and design specific therapeutic targets, but the use of patient-derived cells such as fibroblasts is gaining in strength because they constitute platforms to model disease etiopathogenesis and try new therapeutic approaches in the genetic and epigenetic background of the patient.

New challenges and potential developments in the field of PD entail the validation of these novel therapeutic candidates focused on modifying the course of PD through, among others, promoting mitochondrial and autophagic performance.

## Author Contributions

All authors listed have made a substantial, direct and intellectual contribution to the work, and approved it for publication.

## Conflict of Interest Statement

The authors declare that the research was conducted in the absence of any commercial or financial relationships that could be construed as a potential conflict of interest.
